# A hierarchical cluster-based segmentation analysis of potential solid waste management health hazards in urban Ethiopia

**DOI:** 10.4102/jamba.v11i2.716

**Published:** 2019-07-05

**Authors:** Tendayi Gondo

**Affiliations:** 1 Department of Urban and Regional Planning, School of Environmental Sciences, University of Venda, Thohoyandou, South Africa

**Keywords:** Threat, Vulnerability, Human Health, Aquatic Life, Territorial

## Abstract

Many interventions were sought in the past to address the human health and aquatic life implications associated with poor Municipal Solid Waste Management (MSWM) practices. Majority of such interventions failed to recognise that such human health risks and threats to aquatic life are to a large extent moderated by unique characteristics of different urban and rural spaces where such waste is generated. They failed to employ multiple criteria-based evaluation models that are appropriate in depicting the complex and often interrelated criteria inherently associated with MSWM. This study used the Hierarchical Cluster Analysis (HCA) to evaluate several interdependent variables that define human health and aquatic life hazards associated with poor MSWM practices. Specifically, HCA was used to identify relative similarities among, and distances between a sample of 26 Ethiopian cities and towns in terms of MSWM health threats. Results indicated that threats to human health and aquatic life are surmountable for cities whose economies are relatively low and lacking capacity in terms of SWM infrastructure, acceptable institutional arrangements and better health-care facilities to deal with associated SWM-induced human health risks. Risk of flood waters owing to low altitude has also compounded the urban health conditions in such cities. Despite being better positioned, the analysis observed that some bigger cities still face problems in terms of effective land use planning policies, commitment towards implementing effective SWM programmes as well as the absence of water safety management plans. It concluded by proposing a number of targeted interventions seeking to improve the human health conditions of cities failing to cope with uncollected waste.

## Introduction

Many interventions have been sought in the past to address the human health and aquatic life implications associated with poor solid waste management (SWM) practices. Majority of such interventions however have failed to recognise that human health risks and threats to aquatic life associated with poor SWM practices are to a large extent moderated by unique characteristics of different urban and rural spaces where such waste is generated (Zhou et al. 2010). More often, they have failed to employ multiple criteria-based evaluation models that depict complex and interrelated criteria usually associated with municipal solid waste management (MSWM) (Liao & Chiu 2011). Most studies on MSWM have characterised waste management as a quasi-linear process that more often than not fails to depict important SWM interrelationships that hold the key to developing effective waste management strategies (see Xu et al. 2003; Zhou et al. 2016).

Research and practices associated with solid waste have largely remained uninformed by appropriate territorial studies that recognise the uniqueness of different urban and rural spaces and their associated variations in solid waste-induced threats to human health and aquatic life. Envisaging waste management as a complex and challenging process that is largely influenced by territory- or context-specific attributes has been identified as an important component in developing informed SWM policies (Ripa et al. 2017). Although Ripa et al. (2017:1) caution against performing streamlined and generalised analyses, they also acknowledge the need to identify ‘…context specific waste hierarchies and priorities’.

This analysis therefore seeks to identify and analyse territorial attributes that are hypothesised to result in a diversity of SWM scenarios and their associated threats to human health and aquatic life. This analysis argues that insights into the diversity of SWM spaces and their resultant threats to human health and aquatic life form a starting point for mapping intervention strategies and achieving the Sustainable Development Goals (SDGs) on sanitation. It further argues that such analysis if carefully conducted will permit the identification of criticalities and improvement potential towards new SWM management strategies.

The article is built up as follows. Building on existing SWM, vulnerability and territorial studies literature, the next section presents the analytical framework and methodology utilised for gathering and interpreting the data obtained. Results are then presented and discussed in the next section. The article winds up by giving some concluding remarks.

### Territorial attributes and solid waste management threats: The conceptual framework

The effects of waste and bad waste disposal practices are well documented. Solid waste that ends up in waterbodies negatively changes the chemical composition of the water. This affects the ecological integrity of water ecosystem, which in turn has far-reaching health impact on both humans and aquatic life, whose life is dependent on the availability of clean water (Black 2001; Langergraber & Muellegger 2005). It can also cause harm to animals that drink such polluted water. Some studies have observed that organic wastes discharged from urban communities have far-reaching global and regional ecological and health impacts (Zhou et al. 2010) – which by extension may require a much more global analytical perspective that will help inform context-specific waste management practices. Specific health problems associated with organic wastes, which are usually transmitted through contamination of water and food, include diarrhoea, hepatitis and cancer (Winblad & Hebert 2006; Xu et al. 2003).

**FIGURE 1:**
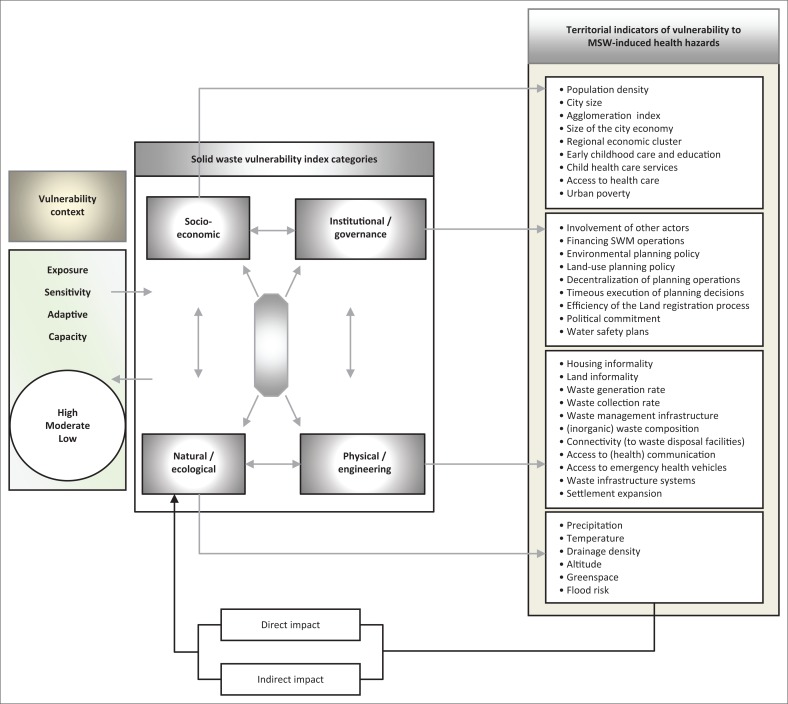
The proposed municipal solid waste management vulnerability assessment framework.

Conceptualising such and other solid waste-related health implications will certainly require linking specific territorial attributes to associated SWM threats. To assess the vulnerability of urban communities to both predictable and unpredictable municipal solid waste (MSW)-induced health hazards, the analysis developed a vulnerability assessment framework (VAF) as an analytical tool premised on complex interaction of human and natural indicators. The proposed VAF assumed that MSW-induced threats to human health and aquatic life in a particular urban territory can reasonably be approached from a set of (territory) behaviour-related variables that subsequently define a sequence of activities depicting a particular pattern in the behaviour of that territory. Specifically, the exposure of urban communities to predictable and unpredictable MSWM-induced health hazards is assumed to be driven by a more complex interaction along physical, natural, institutional and socio-economic factors that produce varying MSWM health hazard outcomes. Such an assumption is consistent with the works of Mose (1998) and Antwi et al. (2015). In principle, it is possible to identify and develop indicators at specific urban localities and assess vulnerability of communities to solid waste-induced threats based on the institutional, socio-economic, engineering and ecological settings of localities (Figure 1).

The main components of the proposed VAF are generally consistent with the thinking behind related studies in waste management. Oppio and Corsi (2017), for example, have performed a territorial vulnerability analysis of local conflicts associated with waste disposal siting in the Lombardy region of Italy. In this and other related efforts, a vulnerability index (VI) that is constant with the three main dimensions of sustainability (environmental, social and economic) was developed (Oppio & Corsi 2017; Oppio et al. 2015; Toro et al. 2012).

**FIGURE 2:**
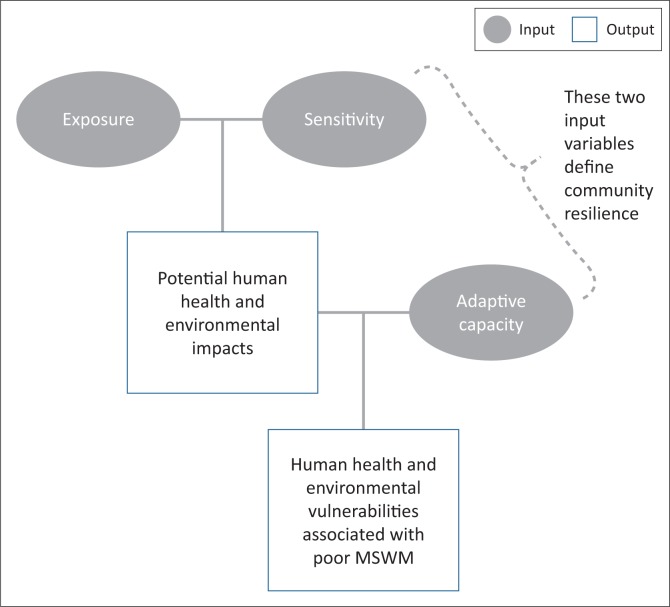
Vulnerability context variables defining municipal solid waste management-induced human health risks.

The proposed VAF assumes that the human health vulnerabilities associated with MSWM are a function of three variables including exposure, sensitivity and adaptive capacity. The exposure component is not misplaced because a significant number of MSWM studies and proposed interventions have in the past emphasised the importance of reducing exposure of communities to poor SWM practices (Arup 2016; Gondo et al. 2010). A host of other studies in the similar field have also sought to understand responses and tolerances (here referred to as sensitivity) of communities to MSWM services rendered by planning authorities (Chandak 2010; Medina 2010). Adaptive capacity in the literature on MSWM has been conceptualised and operationalised in two ways. One stream of research has extensively looked at the extent to which communities are able to cope with changes in MSWM practices mostly brought about by changes in urbanisation and economic development settings in which such communities are situated (Johnson et al. 2016; Ripa et al. 2017). Another strand of research scholarship has looked at the ability of urban communities to cope with or influence changes in prevailing MSWM practices (Cameron et al. 2012; Panagopoulos et al. 2016). Potential human health impacts that subsequently define the extent to which different urban communities are susceptible to poor MSWM practices can therefore in principle be characterised as an output variable that is shaped by the three input variables including exposure, sensitivity and adaptive capacity (Figure 2).

The indicators proposed in this study, although not exhaustive, are deemed adequate for operationalising the concept of MSW-induced vulnerability. Their selection is based on a critical review of literature on MSWM and related environmental and human health concerns. Critical connections that exist between such indicators and MSWM vulnerabilities are discussed in the following sections.

## Engineering attributes defining municipal solid waste management vulnerabilities

The engineering component of the proposed VAF builds on the main theoretical concepts associated with engineering and physical vulnerabilities. Engineering vulnerability in the context of this analysis assesses how much prone a system’s ability to return to steady state is after disturbance event that affects the human health and environmental integrity of that system (Johnson et al. 2016). A steady state in this case can be thought of as ‘effective and efficiently managed waste’, although a disturbing event can be any engineering or physical planning-related activity or process resulting in poor MSWM practices. Related variables are discussed below.

### Planned and unplanned settlements

The proportion of households living in unplanned settlements and their relative location to surface and underground waterbodies is worth investigating, as it has obvious implications on the amount of solid waste that is generated and that eventually finds its way to both surface and underground water sources. Spatial planning and land management in planned communities help reduce the transfer of uncollected waste material in contaminated landscapes to human beings by identifying and isolating areas where health risks are deemed to be high and unacceptable (Cameron et al. 2012; Pacione 2003; Panagopoulos et al. 2016). Unplanned settlements on the other hand can lead to an unhealthy environment characterised by indiscriminate disposal of waste materials (Brown 2003; Brown & Jameton 2000; Chiesura 2004).

### Uncollected waste

The global, regional ecological impacts and localised environmental quality impacts of uncollected waste are well documented (Nicolli et al. 2010; Zhou et al. 2016). Improperly disposed wastes result in health problems, such as diarrhoea, hepatitis and cancer, by means of contaminating water and food (Xu et al. 2003; Winblad & Hebert 2006). It has also been argued that improper handling of MSW could cause a serious damage to ecosystem services by increasing water, soil and air pollution (Mor et al. 2006; Rodríguez 2002). Other scholars also believe that it may also increase the probability of serious impacts on public health (Al-Khatib et al. 2010; Liao & Chiu 2011) or human safety (Mor et al. 2006).

### Solid waste management infrastructure

Availability of basic SWM infrastructure and other innovations associated with landfills is critical in achieving sustainable SWM. Empirical evidence from elsewhere shows that in low-income countries, more than 50% of the collected waste is often disposed of through uncontrolled landfilling and about 15% is processed through unsafe and informal recycling (Chalmin & Gaillochet 2009). Instead of just trying to cope with ever increasing amounts of waste through treatment and disposal, cities are also encouraged to explore other innovative practices such as pursuing best SWM practices that seek to reduce, reuse and recycle (3Rs) waste in a manner that places highest priority on waste prevention, waste reduction and waste recycling (UN-HABITAT 2009).

### Health care access and infrastructure

Communities with poor access to health-care infrastructure and services are more likely to suffer the human health risks associated with poor MSWM practices. Gunn (2009) and the UN-HABITAT (2009) have extensively reviewed some of these challenges including:

- A significant increase in the incidence of sickness among children who live in households where waste is dumped or burnt in the yard.
- Uncollected solid waste usually clogs drains resulting in flooding and subsequent water-borne diseases.
- People living on the wind-ward side of a burning dumpsite will likely suffer from respiratory diseases.
- Contaminated liquids or leachate, leaking from dumpsite, could pollute drinking water supplies.
- Waste dumps potentially serve as breeding ground for Malaria (Gunn 2009; UN-HABITAT 2009).

All the above and other risks require the availability of health care facilities and associated health infrastructure.

### Connectivity

Roads promote transport connectivity by facilitating the easy transportation of SW generated (MUDHCo 2015). Irregularity in the delivery of door-to-door collection and accumulation of solid waste at temporary solid waste collection sites by municipalities, the private sector operators and other actors have been associated to a large extent with the availability and condition of access roads (MUDHCo 2015:72). Where poor road connectivity has resulted in municipalities failing to offer waste collection services, the affected communities have often responded under the guidance of the Not In My Back Yard (NIMBY) Syndrome (Oppio & Corsi 2017). This practice alone has had serious environmental consequences or posed potential risks to human health (Buffoli et al. 2012; Capolongo et al. 2011).

### Socio-economic attributes defining municipal solid waste management vulnerabilities

This component of the proposed VAF is premised on the understanding that some urban communities are more susceptible to the adverse effects of poor MSWM practices because of the socio-economic setting that characterise them. Following the cue from Adger and Kelly (1999) and Eidsvig et al. (2011), it can be argued that the ability of urban communities to cope with and adapt to any external stress placed on their human health and environment by known or unforeseen changes in MSWM practices is largely a function of the socio-economic setting of that community. Such factors are discussed below.

### Urbanisation and other related factors

Liao and Chiu (2011) have noted that waste disposal volume is typically proportional to the growth of population and that associated impacts and risks are in turn more serious in larger cities with higher population densities than in smaller cities. Appropriate waste management practices should therefore take cognisance of the implications posed by population densities and city size as these are associated with certain levels of environmental and human health concerns (Liao & Chiu 2011; Ripa et al. 2017; WHO 2006).

### Level of economic development

Available data in developing countries have shown that there is a positive correlation between the generation of MSW, wealth (Gross Domestic Product [GDP] per capita) and urbanisation (Modak 2011; UNEP 2011; Veolia Environmental Services 2006; World Bank 2005). The bigger the urban economy, the greater the amount of waste generated and consequently the greater the challenge of managing that waste.

### Poverty

Like in many other environmental problems, the poor are the ones who suffer most as a result of improper SWM (Consortium for Research on Educational Access, Transitions and Equity [CREATE] 2010). The largest concentration of the urban poor is also not good for revenue generation. The willingness to pay for SWM is reportedly higher among the rich as compared with the poor. Poor urban residents, particularly those who occupy areas that lack refuse collection, usually dump their waste at the nearest vacant lots, public space or river, or simply burn it in their backyards (Medina 2010).

### Education and awareness

The significance of early learning in raising general awareness of hygiene is obvious. The problem of MSWM is arguably more in many underdeveloped African countries because of a lack of education and awareness (Sharma et al. 2011). The ‘throw away’ garbage attitude that is promoted by the NIMBY syndrome is systematically linked to lower levels of education and awareness among communities where poor MSWM mechanisms exist (Buffoli et al. 2012; Oppio & Corsi 2017).

High levels of education and awareness are also critical in changing the attitude of urban communities towards waste. Where a sizable proportion of urban communities including the municipal leaders who have high levels of education are highly aware of the problems of MSW, public information campaigns that encourage the reduction of waste by turning what used to be seen as ‘waste’ into what can be seen as ‘resources’ are likely to be effective (De Troyer et al. 2016; UN-HABITAT 2009).

### Access to clean water

Water scarcity has remained an important global issue affecting the public health of most developing countries (Montgomery & Elimelech 2007). Poor MSWM systems have exacerbated the challenge of lack of access to clean water via increased water contamination. Urban communities where a greater proportion of their residents have limited access to clean water face a much greater risk of accessing water from contaminated water sources (De Troyer et al. 2016; WHO & UNICEF 2015).

## Institutional attributes defining municipal solid waste management vulnerabilities

The institutional and governance component of the proposed VAF relates to issues pertaining to existing institutional support systems and structures and the right to participate in decision-making process at any level including local, regional, national or international (Turner & Dumas 2013). The major assumption, which is also borrowed from institutional vulnerability literature (see Turner & Dumas 2013), is that the absence of appropriate institutional support systems, structures and processes in some urban localities has exposed their communities to more adverse human health and environmental consequences associated with poor MSWM practices. Critical variables are discussed below.

### Actor arrangements in solid waste management

Traditionally, SWM responsibilities have been the prerogative of the state. Such a one-size-fits-all model has since been replaced by multiple approaches to SWM in which a number of other actors including the private sector and the voluntary sector play a part. Such a migration from the traditional, centralised SWM system to a decentralised system is viewed to be necessary as most governments have often found it difficult to implement effective systems of SWM owing to resource limitations (Letema et al. 2014; Oberlin 2011). Understanding the extent to which urban spaces have moved away from a one-size-fits-all approach to multiple and much more decentralised approaches to SWM is therefore crucial.

### Decentralised planning operations

A paradigm shift from the much more centralised waste management operations to decentralised ones is essential for cities to effectively manage waste. Decentralised frameworks offer an enabling environment for effective and efficient SWM systems. Command and control approaches alone will not improve waste management practices and therefore need to be supplemented by market-based instruments with incentives and disincentives targeted at stimulating investments and entrepreneurship in waste management (Medina 2010; UN-HABITAT 2009).

### Political commitment and financing municipal solid waste management operations

Well-resourced municipalities are expected to promote sound SWM systems associated with limited human health and environmental consequences (Chandak 2010). Where such resources are thin, the consequences thereof are unimaginable. Such an observation has been confirmed by the United Nations University – World Institute for Development Economics Research (UNU-WIDER 2010). Associated with SWM finance is the need for political will. UNESCO (2011) has maintained that a lack of political commitment is widely cited as a reason for the slow progress in the implementation of many municipal programmes.

### Land use planning and environmental policy

It has been established earlier on that planned settlements are characterised by a sound MSWM system (Cameron et al. 2012; Panagopoulos et al. 2016), which, to a large extent, benefits from a sound land use and environmental planning policy. A sound land use and environmental planning policy framework encourages the efficient utilisation of resources. Efficient use of resources can lessen environmental burdens at the local scale such as urban air or water pollution, floods induced by solid waste clogging drainage canals and reduced availability and quality of freshwater supplies (Sameshima 2009).

## Ecological attributes defining municipal solid waste management vulnerabilities

Available literature shows that certain ecological or rather natural variables are intricately related to different MSWM regimes. Subsequently, the nature and extent of such relationships explains why some urban communities are potentially more vulnerable to human health and environmental threats associated with MSWM (De Lange et al. 2010). A host of such factors are discussed below.

### Climate variability

The general observation is that MSWM threats to human health are high where climate change and variability result in high water tables (Bates et al. 2008; Loaiciga 2003) and subsequently exposing uncollected solid waste to flood waters. The vulnerability of water quality to climate change and climate variability is however not a linear process but rather a process that is associated with complex, nonlinear climate feedbacks (Green et al. 2007). High precipitation levels associated with global warming in some regions have meant increased air pollution as excess moisture comes into contact with uncollected waste. Where waste is moist, it has a tendency to generate odours which are a nuisance to the health of human beings (Panagopoulos et al. 2016). A study by Gondo et al. (2010) also showed that the adverse effects of uncollected solid waste are insurmountable in areas characterised by high precipitation levels and high temperatures.

### Waterbodies

The extent to which a particular urban landscape is dissected by waterbodies is critical to understand. State of Ethiopian Cities Report (2015) has noted that in 27 cities of Ethiopia, rivers are virtually used as effluent discharging points for both residential units as well as manufacturing industries. This results in detrimental public health-related hazards to those residing in downstream locations and who use river water for domestic as well as agricultural purposes. Water pollution therefore is a major environmental concern, particularly in those urban landscapes that are located near large waterbodies including rivers, lakes and wetlands (MUDHCo 2012).

### Greenspace

It is argued that the presence of well-designed and well-managed green spaces in inhabited areas is critical to the well-being and quality of life in urban landscapes (Panagopoulos et al. 2016). The general understanding is that urban green spaces such as parks, forests, streams and gardens decrease environmental pollution (Wolch et al. 2014) and subsequently contribute to the quality of the air (Medina 2010).

## Materials and methods

The foregoing literature has showed that a multitude of criteria need to be considered for analyses, if we are to accurately identify territorial clusters that are associated with specific SWM threats to human health and aquatic life. Cluster analysis is one among several other approaches that can help in handling several interdependent variables and criteria associated with MSWM consequences in a better sensible and logical manner. Other approaches range from traditional and often rigid approaches such as the hierarchical structure and multi-criteria decision-making approach (Tseng & Lin 2009), the Decision Making Trial and Evaluation Laboratory approach reported in Liao and Chiu (2011) to flexible ‘linear-in-parameters multivariate statistical modelling technique’, such as Structural Equation Modelling (SEM) (Sarkis 2003; Tseng et al. 2008) and Interpretive Structural Modelling (ISM) (Agarwal et al. 2007; Warfield 1974). As this analysis sought to develop a hierarchical framework that is sufficiently general and could easily be applied in identifying important clusters depicting cities and towns affected by common SWM threats to human health and aquatic life, hierarchical cluster analysis (HCA) was chosen.

### Study area

**FIGURE 3:**
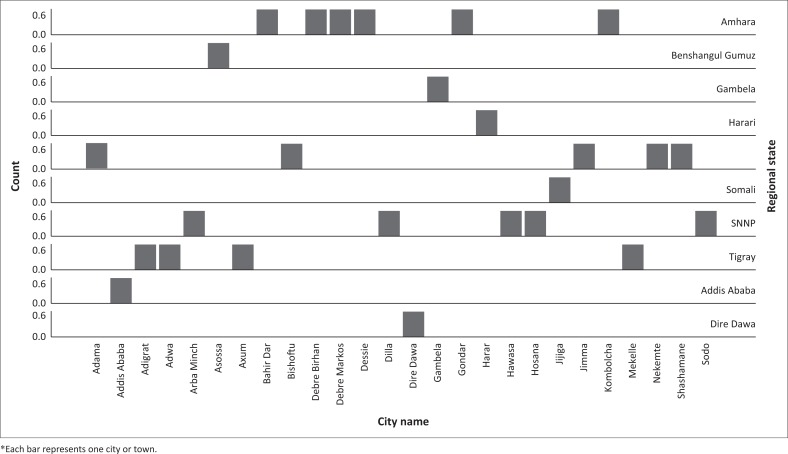
Number of sampled cities per region.

The study was confined to a sample of 26 Ethiopian cities and towns, where a reasonable amount of data on study variables was present (Figure 3).

### Hierarchical cluster analysis explained

Few studies have attempted to examine large urban data sets and to conduct comparisons that identify unexpected similarities and differences among MSWM scenarios defined according to health consequences thereof. This analysis uses HCA to identify relative similarities among and distances between a sample of 26 Ethiopian cities and towns in terms of MSWM health threats. HCA refers to a class of multivariate statistical techniques developed for the analysis of data collected from dependent groups or clusters. HCA allowed the analysis to consider the relationship between MSWM predictor variables and associated outcomes as defined by their effect on human health. Conceptually, the analysis denotes associated health outcomes for MSWM variable *i* in city *j* as *Y*_*ij*_. This outcome is represented in Equation 1 as a function of the individual characteristics, *X*_*qij*_, and a model error *r*_ij_ (Bryk & Raudenbush 1992):

$$Y_{\text{ij}}=\beta_{0\text{j}}+\beta_{1\text{j}}X_{1\text{ij}}+\beta_{2\text{j}}X_{2\text{ij}}+\ldots +\beta_{\text{nj}}X_{\text{nij}}+r_{\text{ij}}$$ [Eqn 1]

where *r*_*ij*_~*N*(0,σ^2^).

The HCA approach adopted here is essentially a bottom-up process, where objects and then clusters of objects are progressively combined on the basis of a linkage algorithm that uses the distance measures to determine the proximity of objects and then clusters to each other (Legendre & Legendre 1998). The analysis adopted ‘Euclidean distance’ as a standard metric to calculate distances (interpreted as the similarity) between all objects in a data matrix (Olden et al. 2012). This was performed on the basis that those objects (i.e. Ethiopian cities and towns) closer together are more alike than those objects that are further apart. An acceptable solution was achieved using Ward (minimum variance) distances. The basic Euclidean distance formula was used as there were no theoretical reasons to prefer a more complex formula, and other formulas did not produce substantially different or more interesting results. At each step, the pair of clusters merged was based on the optimal value of the error sum of squares as defined in Equation 2.

$$d_{\text{ij}}=d\{X_{\text{i}}\},\{X_{\text{j}}\}={\|X_{\text{i}}-X_{\text{j}}\|}^{2})$$ [Eqn 2]

where *d*_*ij*_ is the squared Euclidean distance between *X*_*i*_ and *X*_*j*_.

The results of hierarchical clustering were also visualised using a tree-like structure known as a *dendrogram*. A Geographical Information Systems (GIS) map using arcview GIS was constructed to give a spatial visual of the results. Testing for the significance of the resulting cluster was completed using Mann–Whitney *U* test.

### Variable measurement criteria and data source

This analysis is largely based on the secondary data collected from central sources and administrative records kept by city-level offices in each sampled municipality. The coverage of the data was not always as desired as not all administrative records kept by the cities were up to date with all the required information. In addition, the analysis also relied on the availability of secondary data already documented in connection with the donor-supported programmes and projects implemented that could be readily accessed. City comparisons could only be made on variables for which reliable and comparable data could not be obtained.

The current state of affairs regarding the data environment, namely the capture, storage and sharing of urban data for Ethiopian cities and towns, in some instances, would not permit data extraction on some study variables – an attribute that has been noted in MUDHCo (2015). Although the affected variables were not selected for the analysis, inferences from regional statistics and in some cases from published individual city studies were drawn. To guard against making obvious errors, Likert scales that utilised the attributes of interval estimates were used to capture city statistics instead of actual city statistics that would normally portray point estimates – which could be either correct or incorrect. The use of a scoring system is highly recommended in most vulnerability-related studies (Johnson et al. 2016; Pecl et al. 2014). The size of the Likert scales used in this analysis was based on the availability of data and the researcher’s own judgement.

Before cluster analysis was performed, the study constructs were tested for normality. As the study used a multi-item measurement of the same variable, internal consistency had to be measured using standardised scales (Trobia 2008). Normality was tested using measures of skewness and kurtosis although internal consistency analysis employed the Cronbach’s alpha statistic.

### Ethical considerations

In carrying out the study as well as in disseminating the research findings, the author declares that all ethical issues in research were addressed and that there has been no conflict of interest.

## Results and discussion

Before HCA was conducted, data were tested for normality. This is because many of the statistical procedures associated with HCA are based on the assumption that the data follow a normal distribution. The analysis used the commonly employed indices generated from D’Agostino skewness test and Anscombe-Glynn kurtosis test. Such tests were supplemented by results from Lilliefors corrected (K–S) Kolmongorov–Smirnov test (Elliott & Woodward 2007) as shown in Table 1.

**TABLE 1:** Normality and reliability of study constructs.

Study construct	Number of items	Normality tests			Scale reliability and analysis***
		Skewness*	Kurtosis*	K–S test statistic**	Cronbach’s alpha (≥0.6)
Engineering	7	0.967	1.900	0.470	0.619
Socio-economic	4	0.466	0.037	0.114	0.609
Institutional	4	−0.820	−0.280	0.167	0.660
Natural	4	0.316	0.163	0.144	0.425****

* , Assessment of asymmetry and kurtosis analysis used indices for acceptable limits of ±2 (Field 2009; Gravetter & Wallnau 2014; Trochim & Donnelly 2006).

** , df = 26; *P* > 0.05 (with Lilliefors significance correction).

*** , Levels of acceptance are according to Hair et al. (2009).

**** , Because of the relative importance attached to natural factors, some variables could not be screened off from the analysis.

A series of variables were screened from the overall analysis as they failed to satisfy the required level for centrality and reliability. For the physical or engineering study constructs, housing informality, waste collection and settlement expansion were excluded from the analysis. Socio-economic variables that were also excluded include the agglomeration index, regional economic cluster, access to health, child health care services, urban poverty and access to clean water. Institutional variables that were excluded were financing of SWM operations, involvement of other actors in SWM, decentralisation of planning operations, timeous execution of planning decisions and land registration system for improved SWM. Finally, the ecological variables that were eliminated in the final analysis were Greenhouse Gas (GHG) emissions and temperature. The analysis did not view this as a limitation as such variables were already constituted in other variables. Such related or proxy variables are summarised in Table 2.

**TABLE 2:** Removed variables that are constituted in the analysis.

Study construct	Number of variables before screening	Number of variables after screening	Variables removed from analysis	Related or proxy variables remaining in the analysis
Engineering or physical	11	7	Housing informality	Land informality
			Waste collection	Waste generation rate
			Settlement expansion	City size^a^
Socio-economic	9	4	Agglomeration index	Population density and city size
			Regional economic cluster	Size of city’s economy
			Access to health	Child care services with the region
			Child health care services	Child care services within the region
			Urban poverty	Size of city’s economy
			Access to clean water supply	Water quality management plans or water safety plans^a^
Institutional or governance	9	4	Financing of SWM operations	SWM infrastructure^a^
			Involvement of other actors	Political commitment towards regional and local programs
			Decentralisation of planning operations	Political commitment towards regional and local programs
			Timeous execution of planning decisions	Political commitment towards regional and local programs
			Land registration system for improved SWM	Land use planning policy
Natural or ecological	5	4	Temperature	Altitude

SWM, solid waste management.

a , Variable constituted in a different study construct.

**FIGURE 4:**
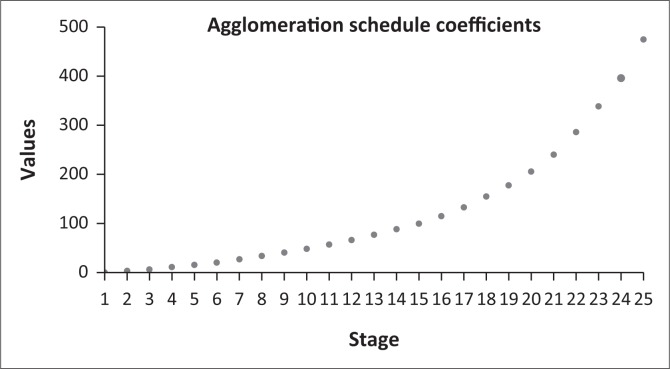
Agglomeration curve. As the ‘step of elbow’ shown by a big black dot appears to be at case number 24, a two-cluster solution should be used (i.e. 26 – 24 = 2).

A two-cluster solution was generated (Figure 4). Because scale variables were used in this analysis, it is a common practice to normalise all the variables before clustering. This is because all clustering algorithms use a distance measure of some sort to determine if object *i* is more likely to belong to the same cluster as object *j* than the same cluster as object *k*. These distance measures are naturally affected by the scale of the variables. The diverse of scales used in this analysis were standardised using *z* scores before clustering was performed. Because the correlation structure in HCA is based on the assumption that data are correlated with a group or cluster, but independent between groups or clusters, checking the relative stability of the final clusters is a requirement (Cameron, Gelbach & Miller 2008). The Mann–Whitney *U* test was used to evaluate the relative stability of the cluster system by testing the hypothesis that the distribution of rank scores was the same across the two cluster solution. The Mann–Whitney *U* test results indicated that the two generated clusters were significantly different (*U* = 165.00; *p* < 0.001). As shown in test view results in Figure 7, the HCA model developed had no stability issues.

**FIGURE 5:**
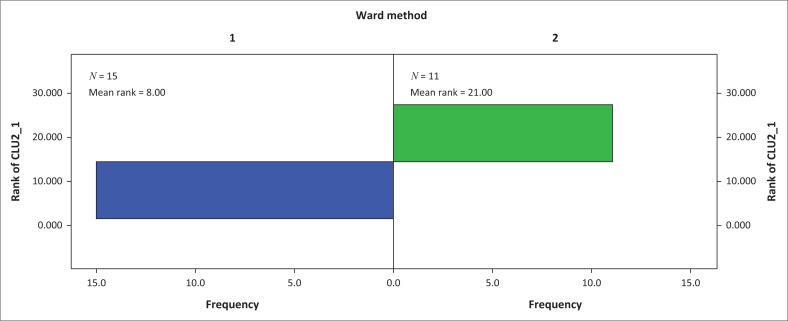
Independent samples Mann–Whitney *U* test.

**FIGURE 6:**
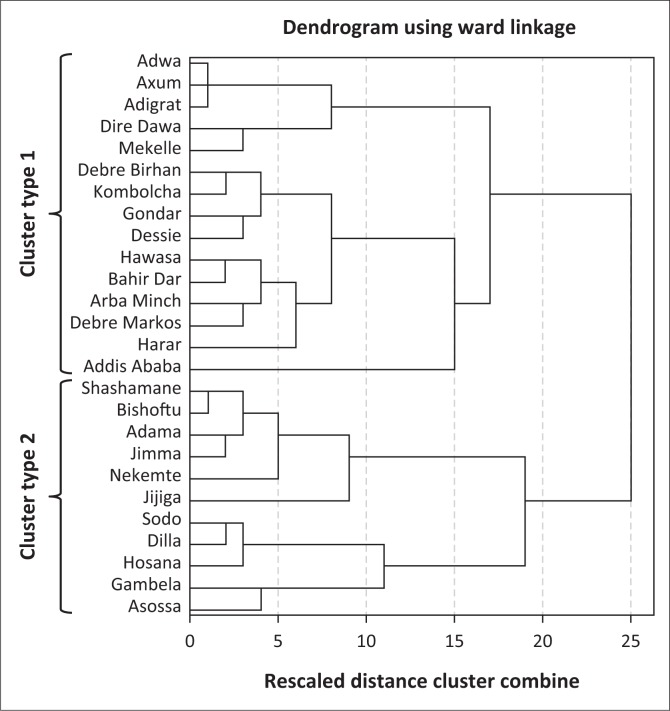
Dendrogram indicating two clusters of cities and towns.

**FIGURE 7:**
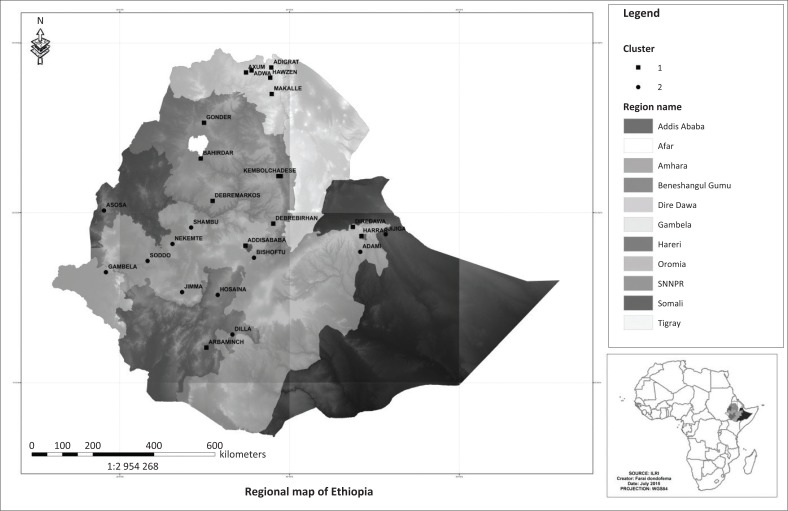
Spatial distribution of cities according to cluster.

Cluster membership results are shown in Figure 6. The spatial distribution of cities according to cluster membership and region is shown in Figure 7. Results indicate that cluster one is the largest with 15 cities exhibiting similar characteristics in terms of MSWM-induced human and aquatic life health implications. The second cluster comprises of 11 cities and towns. In general, both clusters show that Ethiopian cities are constrained in terms of MSWM systems and that this constraint has varying implications on human health and aquatic life. As expected such variations are largely a function of the extent to which each city or town locality is vulnerable to SWM defining engineering, socio-economic, institutional and natural factors – an attribute that is concordant with vulnerability (Antwi et al. 2017; Folke 2006; Mose 1998) and waste management literature (Oppio & Corsi 2017; Oppio et al. 2015; Toro et al. 2012). In terms of the socio-economic variables, cluster one type of cities are in general large and have a better economic development attributes, which have seen them generating large quantities of solid waste.

Although some large cities are characterised by high population densities (e.g. Addis Ababa, Hawassa, Gondar, Bar hirda and Harar), others are characterised by low densities (e.g. Dire Dawa, Mekelle and Dessie). Interestingly, we also see very small cities with low population densities such as Adwa and Axum joining this cluster too. We also observe some smaller cities characterised by high population densities and better economic wealth as measured by city product joining this group. Levels of informality in this cluster are generally high in relative terms although a number of exceptions can be singled out. They generally have a better SWM infrastructure and road connectivity, although exceptions exist (Debre Birhan, Adwa, Axum, Gondar and Dessie). Health care infrastructure is also better in relative terms. The overall institutional standing also looks better in terms of land use planning, environmental policies, political commitment towards implementing programmes as well as existence of water safety management plans. Exceptions however exist as Addis Ababa’s political commitment is constrained. Dire Dawa and Mekelle city have issues with water safety management, and Gondar city has problems associated with the land use planning policy. As shown in Figure 7, the majority of the bigger cities in this category occupy high altitudes and are characterised by high precipitation levels.

Although the situation in all variables looks gloomy for cluster type two cities, some exceptions in terms of cities having better institutional arrangements for SWM exist. These include Jimma and Sheshemane. The majority of cluster two cities are also occupying low-altitude areas, where the risk of flood waters contaminating waterbodies is high. The analysis observes that cluster one type of cities are in relative terms less susceptible to SWM-induced human health and aquatic life threats as they have better institutional arrangements in place, better SWM infrastructure and better health care facilities to deal with associated human health risks. Threats to human health and aquatic life are surmountable to cluster two types of cities whose economies are relatively low and lacking capacity in terms of SWM infrastructure, acceptable institutional arrangements and better health care facilities to deal with associated human health risks. The risk of flood waters owing to low altitude has also meant that uncollected waste will find its way to waterbodies.

## Conclusion

The analysis has challenged mainstream research efforts on MSW threats in Ethiopia that usually characterise SWM challenges as a function of a few variables. Current literature has characterised a few isolated cases of cities and towns. The analysis has managed to take a territorial-based approach that has employed a multiple criteria system to identify unique clusters of cities, where there are potential threats to human health and aquatic life. Although the mainstream literature on MSWM challenges in developing countries holds that bigger cities are more likely to face serious challenges associated with poor waste disposal mechanisms, this analysis has shown otherwise. The analysis has shown that threats to human health and aquatic life are surmountable to medium-sized and smaller cities too, whose economies are relatively low and lacking in capacity in terms of SWM infrastructure, acceptable institutional arrangements and better health care facilities to deal with associated human health risks. Risk of flood waters owing to low altitude has also meant that uncollected waste will find its way to waterbodies of such cities (Tadege 2007).

The results are also consistent with Arup (2016) in the sense that it has developed a VAF similar to Arupe’s Urban Environmental Risk Framework (Urban ER) to help cities to understand and address the critical SWM – health-induced challenges that shape urban well-being. In cities established in low-lying areas such as Adama, Bahir Dar, Dire Dawa and Hawassa (Daniel 2007; MUDHCo 2015), where flood waters risk entering waterbodies, the analysis recommends coming up with effective land use planning, environmental planning policy and, more specifically, effective water safety management plans. Critical SWM infrastructure such as landfills and other innovations should also be introduced to smaller urban centres where the struggle to deal with human health challenges is very high.

Targeted interventions are also recommended in large-sized cities where SWM threats to human health and aquatic life are low in relative terms. In Addis Ababa, concerted efforts should be made to improve political commitment towards implementing SWM programmes. In Dire Dawa and Mekelle city, water safety management plans need to be strengthened and streamlined to effective environmental planning policy. Land use planning challenges that will reduce all forms of informality need to be addressed in Gondar city. Although this analysis may be considered in shaping targeted policy interventions, it also recommends the development of a future risk rating system that will project the future positioning of each city in terms of SWM-induced human health and aquatic life threats.
